# Transport mechanism of deformable micro-gel particle through micropores with mechanical properties characterized by AFM

**DOI:** 10.1038/s41598-018-37270-7

**Published:** 2019-02-05

**Authors:** Wenhai Lei, Chiyu Xie, Tianjiang Wu, Xingcai Wu, Moran Wang

**Affiliations:** 1 Department of Engineering Mechanics, Tsinghua University, Beijing, 100084 China; 2 Department of Petroleum and Geosystems Engineering, The University of Texas at Austin, Austin, Texas 78712 USA; 3 Changqing Oilfield, PetroChina, Xi’an, 710018 Shaanxi China; 4 Research Institute of Petroleum Exploration & Development of PetroChina, Beijing, 100083 China

## Abstract

Deformable micro-gel particles (DMP) have been used to enhanced oil recovery (EOR) in reservoirs with unfavourable conditions. Direct pore-scale understanding of the DMP transport mechanism is important for further improvements of its EOR performance. To consider the interaction between soft particle and fluid in complex pore-throat geometries, we perform an Immersed Boundary-Lattice Boltzmann (IB-LB) simulation of DMP passing through a throat. A spring-network model is used to capture the deformation of DMP. In order to obtain appropriate simulation parameters that represent the real mechanical properties of DMP, we propose a procedure via fitting the DMP elastic modulus data measured by the nano-indentation experiments using Atomic Force Microscope (AFM). The pore-scale modelling obtains the critical pressure of the DMP for different particle-throat diameter ratios and elastic modulus. It is found that two-clog particle transport mode is observed in a contracted throat, the relationship between the critical pressure and the elastic modulus/particle-throat diameter ratio follows a power law. The particle-throat diameter ratio shows a greater impact on the critical pressure difference than the elastic modulus of particles.

## Introduction

Oil and gas still supply most of the world’s energy and extraction of the remaining trapped-oil is significant to meet the demand of future energy^1,2^. Due to the strong reservoir heterogeneity and fluid channeling, it is reported that the normal water flooding can only produce about 30% of the total reserves of oil^3^. To overcome these difficulties, several EOR techniques have been proposed, including polymer flooding^3–5^, oil-in-water (O/W) emulsions^6,7^ and foam displacement^8^. Among these techniques, polymer flooding is considered as one of the most promising technologies^9^. Traditional polymer solutions such as xanthan gums and hydrolyzed polyacrylamides (HPAM) have been widely used in oilfields^4,5^. The high viscous property provide a good mobility control, yet the injection capacity reduction, shear degradation (in high temperature and high salinity) and dilution effect limit their applications. More importantly, these continuous polymer solutions usually do not show promising blocking effects since their small molecular size compared to the pore size^10^.

In recent years, new particle-type polymers, such as preformed particle gel (PPG)^11,12^, soft micro-gel (SMG)^13,14^, elastic microsphere^15,16^, etc., have been developed as a smart sweep improvement and profile modification agent. Micro-gel particle is a network of aggregated colloidal particles with soft solid-like mechanical properties^17^, and its size is comparable to the pore size. The DMP suspension is a viscoelastic fluid in macroscale and the particle is cross-linked as a porous interconnected network structure in microscale. The fact is that DMP is not always effective for all kinds of oilfields in practice^18,19^ due to the unclearness of the transport mechanism of DMP. Understanding the transport mechanism of DMP in micropores of rocks is crucial for gel treatment during enhanced oil recovery.

Experimental studies have presented qualitative observations and measurements of EOR effects of DMP. At macroscopic scale, the core-flooding experiments and sand-packed models^16,20–22^ have been performed to get the fluctuation of the injection pressure and the flow variation. The most important advantages of DMP are blocking the fluid channeling paths and diverting the displacing fluid from higher-permeability layer to lower-permeability layer (so-called diversion effect), which will cause the higher pressure to push the DMP to deform and pass through the pore throats. With the development of microchips, recently micromodel and visualized sand-packed model experiments^11,13,15^ have exhibited several patterns of DMP passing through porous media at pore scale, which have resulted in qualitative correlations with particle diameter, pore throat diameter, particle strength and displacement pressure. Yet quantitative critical conditions are still very difficult to be determined because of limitations of those equipment and experiments. Some recent experiments, such as the capillary flow experiment^23,24^, nuclear-pore film filtration experiments^25,26^ and perforations with different hole-sizes^19,27^ have been conducted and tried to get the relationship between critical pressure and particle pore-throat diameter ratio quantitatively. However, success is few because the microscopic mechanical properties of micro-nano particles have never been characterized.

On the other hand, DMP has been modelled in different scales to understand the transport mechanism in rock pores. Molecular dynamic (MD) simulations^28,29^ were used to study the transport behavior of PPG in nanochannels. However this method is not suitable for DMP transport in complex porous media due to its expensive cost. Zhou *et al*.^30^ simulated PPG deformations through a channel by LBM-DEM method, however, some spring parameters in their model were not determined physically. Xie *et al*.^31^ considered the dispersed system as one continuous non-Newtonian solution and used a two-phase viscoplastic LBM to model the polymer flooding process. Recently, they have developed a three-phase viscoelastic model to further discover the displacing mechanisms of the dispersed polymer system^32^, where the polymer was still regarded as a fluid instead of soft particles. Therefore, when the discrete effects of deformable particles are strong, the above studies are not suitable for the real DMP transport process.

With successful applications of the IB-LBM in red blood cell (RBC)^33,34^ and capsule^35–37^ simulations, it presents a promising way to consider the soft particle-fluid interaction in complex pore-throat geometries. The lattice Boltzmann method (LBM) is a mesoscopic method as the fluid solver. The LBM method is particularly suitable for simulations of fluid flow in a complex geometries, especially the one that is easy to be combined with IB^38^, because of various schemes for the body force being proposed in LBM^39,40^.The immersed-boundary method (IBM) introduced by Peskin^41^ in 1972 has become a popular practical and effective method for fluid-structure interaction problems. Feng *et al*.^42^ were the first to combine LBM and IBM for 2D simulations of rigid disks in 2004. In the IB-LBM system, the fluid is aware of the boundary only through a forcing term. There is no direct boundary condition for the fluid, which means that the populations in LBM can cross the boundaries freely, but the macroscopic fluid behaves as if there was a boundary^38^. This is one of the central ideas of the combined IB-LBM algorithm. The spring-network model^33,43^ has been widely used as the constitutive model of deformable particles. However, the value of each parameter in the spring network model cannot be obtained by physical parameters directly. Although some micromechanical experiments have been carried out for RBC^44,45^, capsules^46^ and emulsions^47^, there is no experiment for the measurements of the DMP mechanical properties yet.

Therefore in this paper, we measure the elastic modulus of DMP by AFM in the nano-indentation experiments primarily to determine the parameters of the spring-network model. Then the IB-LBM framework combining with the spring-network model is applied to study the transport mechanism of DMP in a pore-throat channel.

## Results

### Elastic modulus of DMP

The elasticity of DMP is an important parameter in DMP experiment and simulation. We propose to use AFM to quantitatively measure it in the micro scale. Soft Microgel (SMG) were provided as one of the typical DMPs by Research Institute of Petroleum Exploration & Development of PetroChina^13^, the details of SMG is introduced by Wu *et al*.^13^. In this work, the elastic modulus of the SMG particle measured by AFM-based nano-indentation experiments is 3.22 ± 0.09 MPa, which is calculated by Hertz model, shown in Table 1. The Hertzian elastic contact theory is the mostly used approach with the simplest expression for modeling the tip-sphere contact. The theory has been experimentally demonstrated to be valid for small deformations by a nonadhesive elastic sphere against a plane or sphere^48^. In this work, we don’t consider the size effect of Young’s modulus. The detail of the experiments results is shown in Supplementary Information.

**Table 1:** Elastic modulus of the SMG.

$\bar{E}$(MPa)	*σ*_*A*_	*σ*_*B*_	$\sigma =\sqrt{{\sigma }_{A}^{2}+{\sigma }_{B}^{2}}$
3.22	9.01 × 10^−2^	4.92 × 10^−3^	9.50 × 10^−2^

The standard deviation *σ* has two parts, fitting each nanoindentation curve to get elastic modulus *σ*_*B*_ and combine all elastic modulus to get final elastic modulus *σ*_*A*_.

### Pressure difference variation

**Figure 1:**
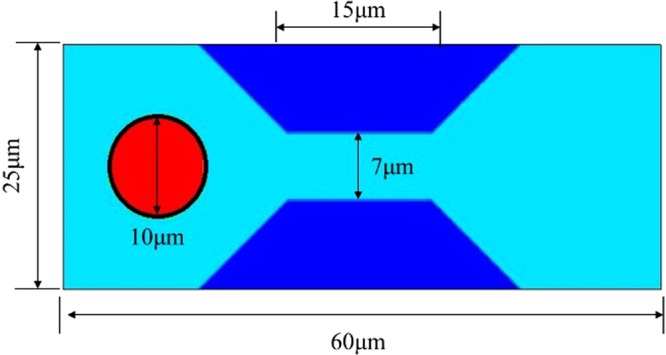
Schematic of a deformable particle gel through a narrow channel. The channel total length is 60 μm, the largest and smallest height of channels are 25 μm and 7 μm, respectively, and the particle-throat diameter ratio is 1.43. The length of the throat part is 15 μm and the length of the transition part equals to the height of the wall.

**Figure 2:**
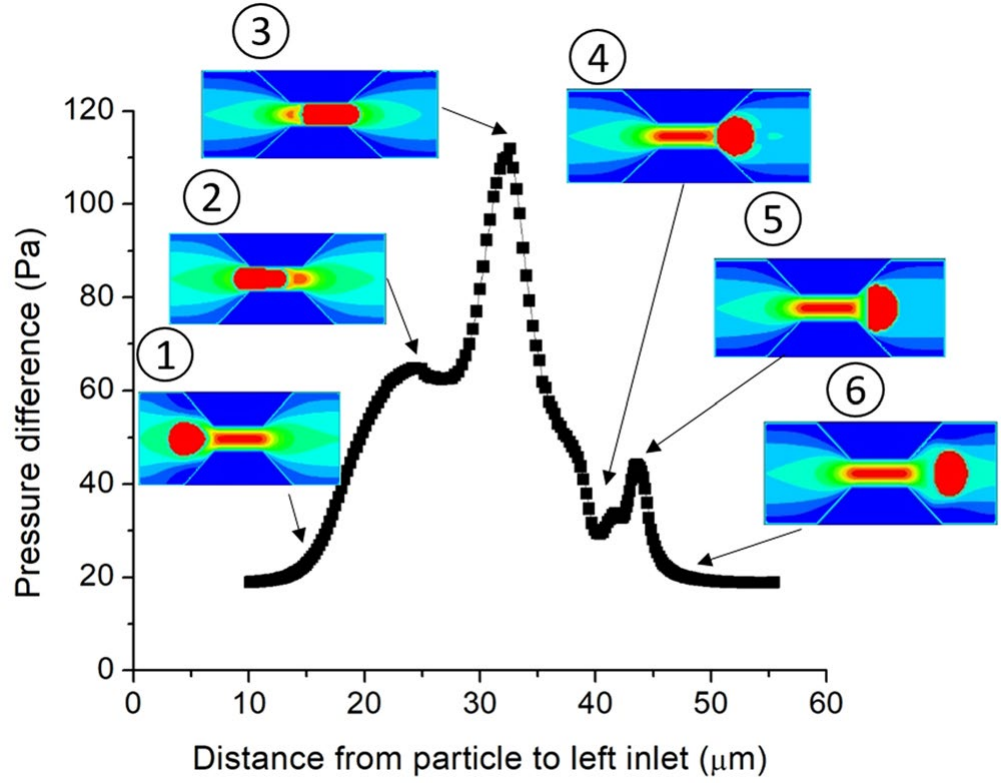
Variation in pressure difference versus particle position in the simulation model.

Correlation between DMP and the reservoirs will affect the final results of gel treatment in EOR. The critical pressure is mainly relied on the particle size, the deformation of DMP and size of the rock pores. Thence, the DMP passing through a contracted throat under different particle-throat diameter ratio and elastic modulus was simulated to understand this correlation. Figure 1 shows the schematic of DMP passing through a narrow constriction. Figure 2 shows the variation in the pressure difference of inlet and outlet channel when the DMP migrated in the narrow pore-throat under constant flow rate (0.001 m/s). The elastic modulus of the simulated particle is 3.20 MPa nearly be the same as the value in the experiment (3.22 MPa) and particle viscosity is 5mPa.s^33^. The no-slip condition of fluid-particle ensure fluid-solid friction, particle-wall friction force is not considered due to this ideal simulation- zero roughness in the wall or soft (not very large stiffness) DMP transport through micropores.

As shown in Fig. 2, the pressure difference relies on the position and the deformability of particle. The pressure difference remains relatively low before the particle reaches the throat (see Fig. 2-stage 1). When the particle enters the throat preliminarily, the pressure difference mainly exists in the slit between particle and wall, shown in Fig. 2-stage 2, and pressure difference reaches the high-pressure difference stage when it goes into the throat completely, shown in Fig. 2-stage 2. However, when the particle is ready to go out, the particle will block the inflection point of the channel width due to the low pressure outside and deformation energy release in the front part of the particle. The pressure difference will increase rapidly, reaching the first peak (see Fig. 2-stage 3). Because of the detachment of the particle to the throat, the pressure difference will decrease rapidly (see Fig. 2-stage 4). There is a transition region between the wide channel and throat, the velocity of the back part of the particle is larger than the front part, its vertical diameter will become large and block the transition part because of the deformation of the particle, the pressure difference will reach the second pick, as shown in Fig. 2-stage 5. Finally, the particle recover to the original shape in the flow field, the pressure difference to the original low level, as shown in Fig. 2-stage 6.

Therefore, we can conclude that the two-clog procession (two peaks of pressure difference) of the DMP transport in a narrow channel will cause the pressure fluctuation in the flow field. There will be two pressure difference peaks during the procession, one for blocking the outside of the narrow channel and one for clogging the transition part of the narrow to wide channel, shown in Fig. 2-stage 3 and Fig. 2-stage 5.

### The effect of particle-throat diameter ratio

**Figure 3:**
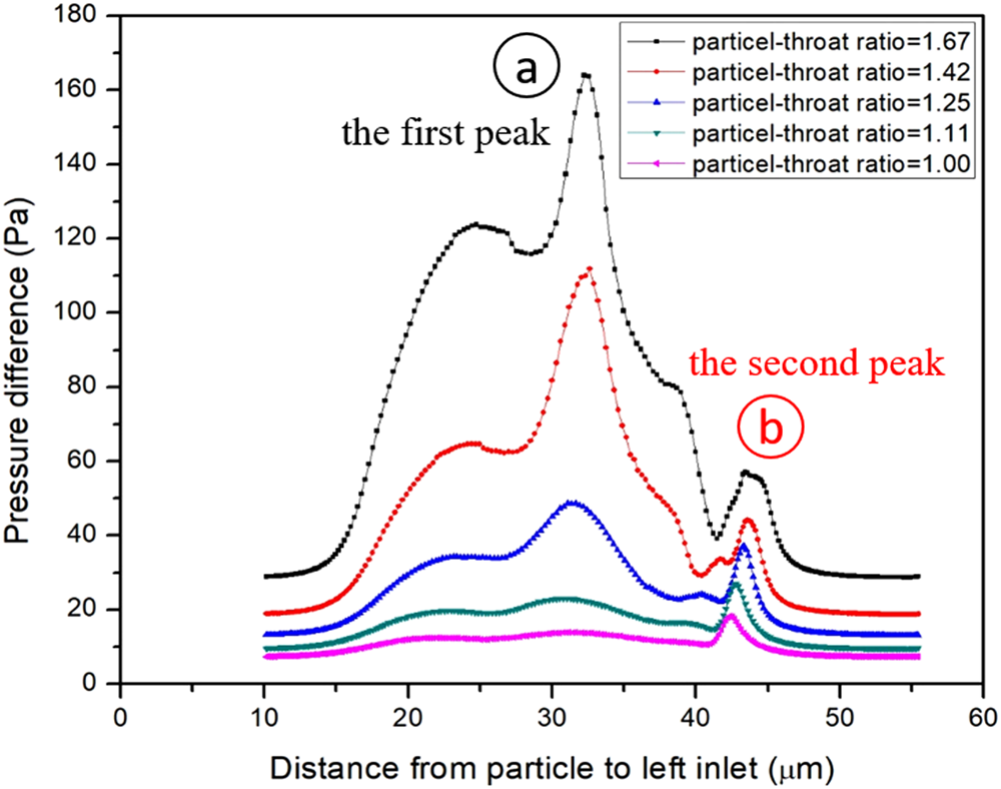
The effect of the particle-throat diameter ratio on the variation of pressure difference. (**a**) is the first peak of pressure difference in the outside of the narrow channel, (**b**) is the second peak of pressure difference in the transition part of the narrow to wide channel.

**Figure 4:**
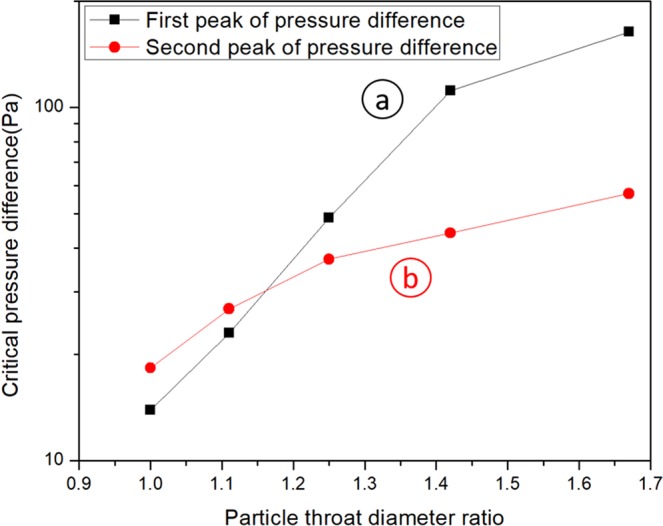
Two peaks of pressure difference in the different particle-throat diameter ratio.

**Figure 5:**
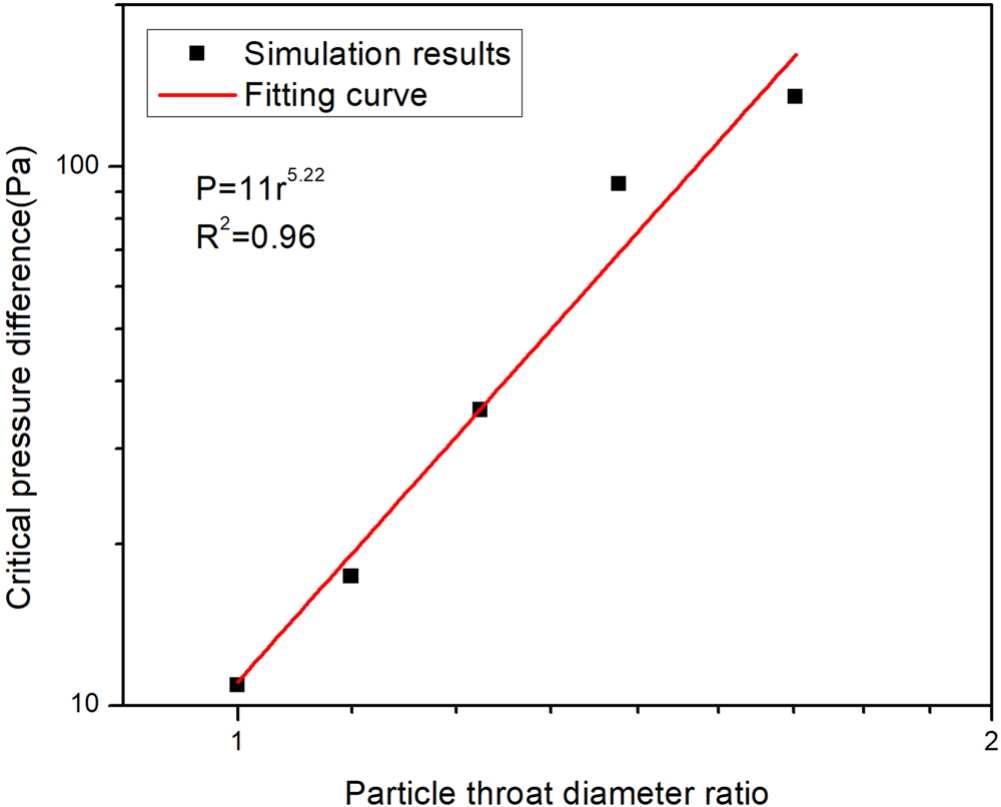
The relationship of the particle-throat diameter ratio with critical pressure difference.

The ratio of the particle-throat diameter *r* is an important factor influencing the blocking effect of the particle in porous media. In this simulation, the height of the throat will change and the diameter of the particle is constant as 10 μm, the particle-throat diameter ratio changes between 1 and 2. In Fig. 3, a higher particle-throat diameter ratio will lead to a larger pressure difference and the particle-throat diameter ratio will not change the blocking position. The pressure differences of these two peaks changing with particle-throat diameter ratio are shown in Fig. 4. When the particle-throat diameter ratio is less than 1.15, the largest pressure difference will happen in the second peak; otherwise, it will happen in the first peak. It means that the blocking effect in channel is really weak, lower than particle dynamic deformation in the transition part. The largest pressure difference with different ratio *r* is plotted in Fig. 5, it can be approximated that the pressure is proportional to the fifth power of the particle-throat diameter ratio.

### The effect of elastic modulus

**Figure 6:**
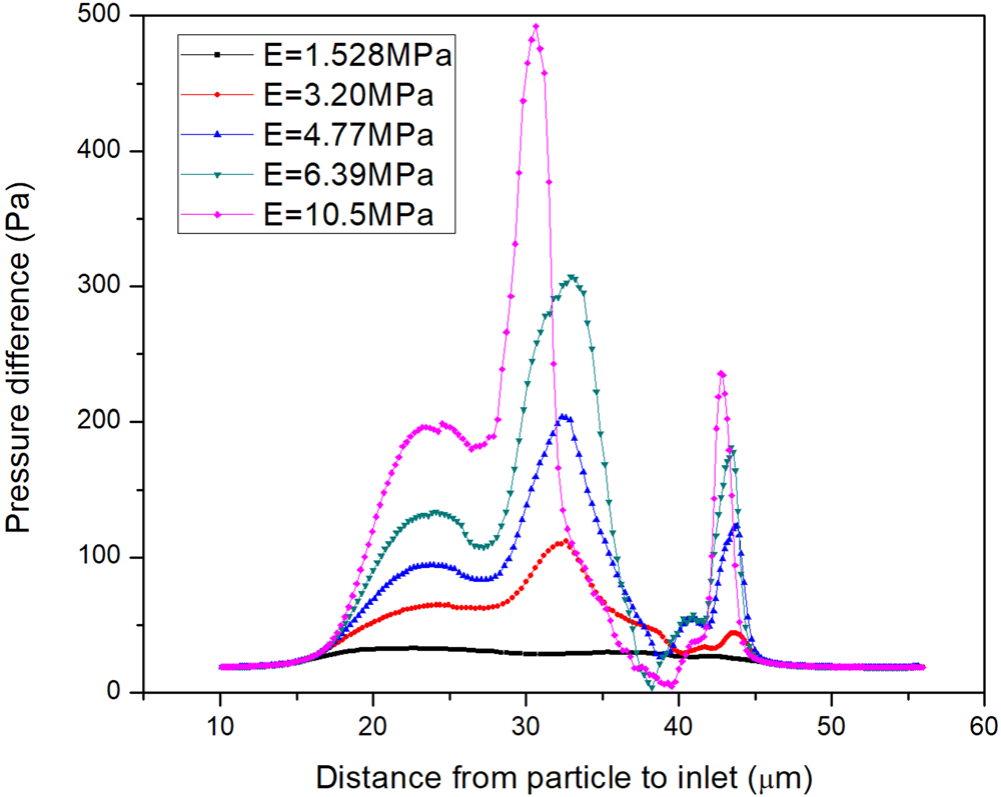
The effect of the elastic modulus on the variation of pressure difference in the different elastic modulus. The particle-throat diameter ratio is fixed on 1.42 and Young’s modulus changes from 1.528~10.5 MPa.

**Figure 7:**
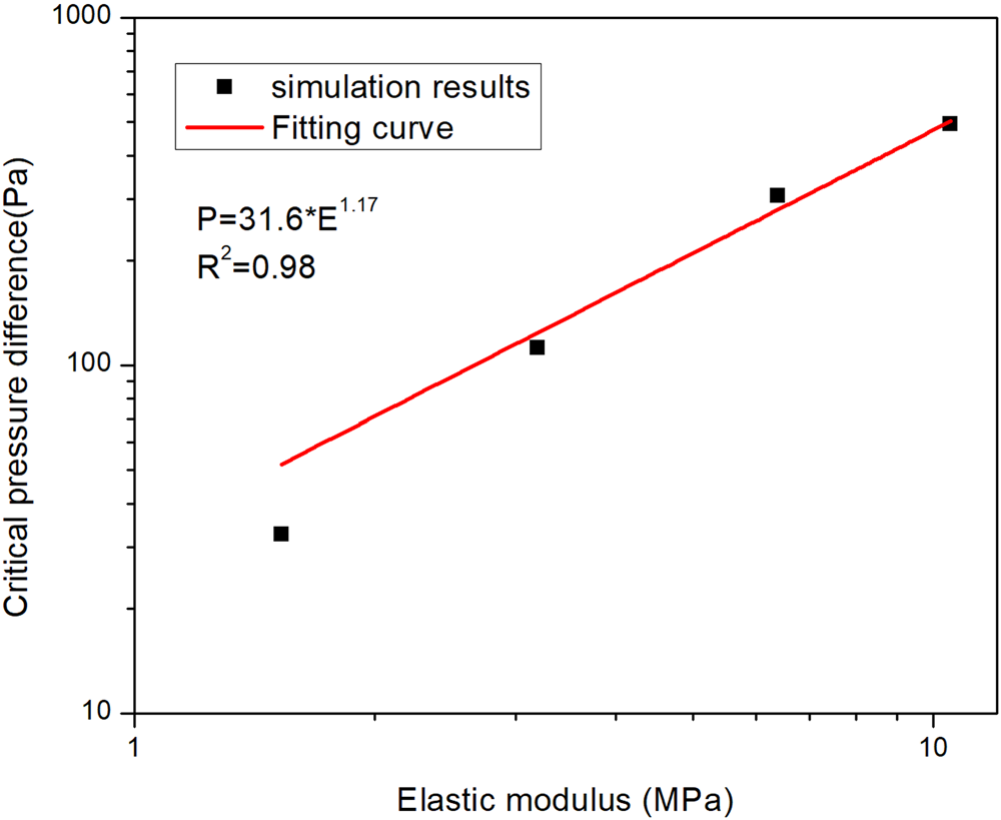
The relationship of the elastic modulus with critical pressure difference.

The effect of the particle deformability is studied by changing the elastic modulus of the DMP model. The effect of the elastic modulus on the variation of pressure difference in the different elastic modulus is shown in Fig. 6. The higher elastic modulus will cause higher critical pressure difference and critical pressure difference will always happen in the first peak. The relationship between critical pressure and elastic modulus is shown in Fig. 7. It approximately shows that the pressure is proportional to the half power of the elastic modulus.

1 $$P\propto {r}^{5.2}{E}^{1.17}$$

On this basis, a relatively clear understanding about the relationship of the critical pressure and elastic modulus is shown in Equation (1), which is an important guide when selecting proper elastic modulus particle and particle size for gel particle treatment for different reservoirs.

## Conclusion

This paper proposes an immersed boundary-lattice Boltzmann framework to study transport behavior and mechanism of deformable micro-gel particles (DMPs) in porous media. The elastic modulus of DMP is measured by AFM-based nano-indentation experiments to provide accurate parameters in the spring-network model for DMP. Based on its intrinsic elastic modulus, we simulate the motion of DMP in a single narrow throat at pore scale, and obtain the following conclusions:

This is the first time to get the elastic modulus of SMG particle by AFM in micro scale. The elastic modulus of SMG particle is 3.22 ± 0.095 MPa, measured in its original state in suspension solution. The spring network model parameters are determined by fitting the particle elastic modulus in nanoindentation simulation. Based on the characterization of particle microscopic properties, numerical simulation is closer to physics.

The two-clog procession is more complex (six stages) than the previous works (clog-deform-pass procession)^24,30^. There are two main peaks of the pressure difference, one for blocking the outside of the narrow channel (see Fig. 2-stage 3) and one for clogging the transition part due to the dynamic deformation (see Fig. 2-stage 5). Particle-throat diameter ratio will influence the relative size of these two peaks, while the Young’s modulus of the particle will not, which means the particle-throat diameter ratio has a greater impact on the particle transport mode. When particle-throat diameter ratio is less than 1.15, the critical pressure difference will always happen in the second peak, which means that the transition part blocking is stronger than the channel blocking, the particle dynamic deformation in the transition part is not negligible.

A power function relationship between the critical pressure and the elastic modulus/particle-throat diameter ratio is acquired. The pressure is proportional to the first power of the elastic modulus and the fifth power of the particle-throat diameter ratio. Therefore, the particle-throat diameter ratio has a greater impact on the critical pressure difference than the elastic modulus. This relationship can be used as an important guide to select proper elastic modulus particles according to various reservoir conditions.

Viscosity of the particle and roughness of the wall are important factors to study transport mechanism of DMP through micropores. However, this work mainly focuses on the particle-throat diameter ratio and particle elastic modulus. In the future work, we will characterize the DMP viscosity and simulate viscoelastic particle transport in narrow channel with roughness.

## Methods

**Figure 8:**
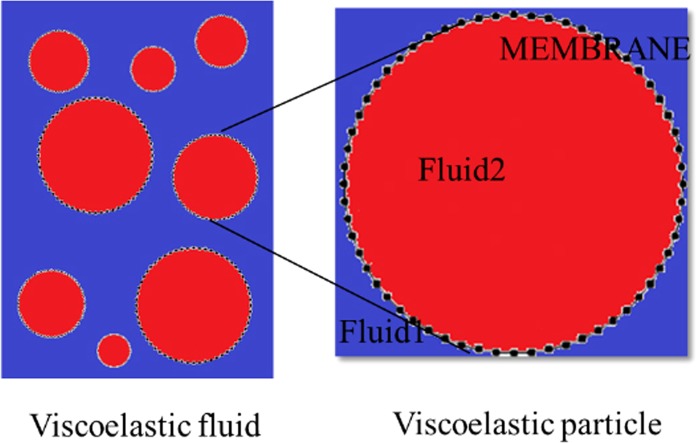
The model for the viscoelastic fluid and the viscoelastic particle. Viscoelastic particle is an important part of the viscoelastic fluid, which will influence the property of the viscoelastic fluid.

A combined IB-LBM method is employed to couple the DMP with the fluid and the spring-network model is used as constitutive model of DMP. The Lattice Boltzmann method is utilized to solve the impressible flow field in a Eulerian coordinate, and an explicit immersed boundary method for the coupling of the fluid and the particle. The response of the particle to deformations is computed by the spring-network model, this is a good method to model viscoelastic particle, as shown in Fig. 8.

### Lattice Boltzmann method (LBM)

The process of individual kinetic particles subject to collision and propagation on a lattice is established by the LBM to recover hydrodynamics on larger scales^38^. The Lattice Boltzmann equation (LBE) with an external force to solve the incompressible N-S equation can be expressed as^39^

2 $${f}_{i}^{\ast }(x,t)={f}_{i}(x,t)-\frac{1}{\tau }[{f}_{i}(x,t)-{f}_{i}^{(eq)}(x,t)]+{\delta }_{t}{F}_{i}(x,t),$$

3 $${f}_{i}^{\ast }(x,t)={f}_{i}(x+{c}_{i}{\delta }_{t},t+{\delta }_{t}),$$

where *c*_*i*_ is the discrete lattice speed; *f*_*i*_*(x*, *t)* is the distribution function for particle with velocity *c*_*i*_ at position *x* and time *t*, and *δ*_*t*_ is the time increment. *τ* is the non-dimensional relaxation time and ${f}_{i}^{(eq)}$ is the equilibrium distribution function.

4 $${f}_{i}^{eq}(x,t)={\omega }_{i}\rho [1+\frac{3{c}_{i}\cdot u}{{c}^{2}}+\frac{9{({c}_{i}\cdot u)}^{2}}{2{c}^{4}}-\frac{3{u}^{2}}{2{c}^{2}}],$$

where *ω*_*i*_ is the weight; *ρ* is the fluid density and u is the fluid velocity; $c={\delta }_{x}/{\delta }_{t}$; *δ*_*x*_ is the lattice spacing; *δ*_*t*_ is the time step.

The forcing term *F*_*i*_ accounts for a body force F and can be expressed as^39^

5 $${F}_{i}=(1-\frac{1}{2\tau })\frac{3({c}_{i}-u)\cdot F}{{c}^{2}\rho }{f}_{i}^{(eq)}$$

The most widely used discrete velocity model is the DnQm model, where n is the spatial dimension, and m is the number of discrete velocity directions. In this paper, the D2Q9 lattice is applied for 2D simulation, defined as

6 $${c}_{i}=[{c}_{ix},{c}_{iy}]=\{\begin{array}{c}[0,0],i=0\\ \{\cos \,[\frac{\pi }{2}(i-1)],\,\sin \,[\frac{\pi }{2}(i-1)]\}c,i=1,2,3,4\\ \{\cos \,[\frac{\pi }{4}+\frac{\pi }{2}(i-5)],\,\sin \,[\frac{\pi }{4}+\frac{\pi }{2}(i-5)]\}\sqrt{2}c,i=5,6,7,8\end{array}$$

7 $${W}_{i}=\{\begin{array}{ccc}4/9,i & = & 0\\ 1/9,i & = & 1,2,3,4\\ 1/36,i & = & 5,6,7,8\end{array}$$

After density distribution function is known, the macroscopic fluid density *ρ*, velocity *u*, pressure *p* and the kinematic viscosity of the fluid *v* are computed as follows:

8 $$\rho =\sum _{i=0}^{8}\,{f}_{i},$$

9 $$u=\sum _{i=0}^{8}{c}_{i}\,{f}_{i}/\rho ,$$

10 $$p=\frac{\rho {c}^{2}}{3},$$

11 $$\nu =\frac{{c}^{2}}{3}(\tau -\frac{1}{2}){\delta }_{t}.$$

### Immersed boundary method (IBM)

The IBM is used to model the fluid-structure interactions. Using body force term to mimic the boundary condition, force spread and velocity interpolation are applied to realize the force and velocity exchange between the fluid and the interface of the particle. The effect of Newton’s third law between moving boundary and fluid is realized by spreading the stress of the nodes on moving boundary onto the fixed Eulerian grid points near the boundary. The velocity of the moving boundary nodes is interpolated from the adjacent fluid nodes with the Dirac function to ensure the no-slip condition.

In IBM, the force spread is the membrane force *f*(*x*_*m*_) at a membrane point *x*_*m*_ is spreading to the nearby fluid grid points *x*_*f*_, through a discrete delta function *D(x)*, which is chosen to approximate the properties of the Dirac delta function^49^.

12 $$F({x}_{f})=\sum _{m}D({x}_{f}-{x}_{m})f({x}_{m}).$$

Subsequently, the velocity of the membrane point *u*(*x*_*m*_) is updated based on the local flow field according to the Euler scheme

13 $$u({x}_{m})=\sum _{f}D({x}_{f}-{x}_{m})u({x}_{f}).$$

The function *D(x)* of Equations (12) and (13) used for force spreading and the velocity interpolation, is replaced by a product of 1D interpolation kernel functions *D(x)*.The so-called 4-point stencil reads^38^

14 $$D(x)=\{\begin{array}{cc}1/8(3-2|x|+\sqrt{1+4|x|-4{x}^{2}}) & for\,0\le |x|\le 1\\ 1/8(5-2|x|-\sqrt{-7+12|x|-4{x}^{2}}) & for\,1\le |x|\le 2\\ 0 & for\,2\le |x|\end{array}.$$

### Constitutive model of deformable micro-gel particle

A spring network model based on the minimum energy principle is applied to express the elastic behavior of the deformable particle. The elastic energy, E = E_l_ + E_b_, is stored in the following two parts, stretch/compression spring energy and bending energy of two neighboring stretch/compression spring elements^33,43,50^.

The stretch/compression spring energy *E*_*L*_ due to the change in length l_I_ from its reference l_0_ is expressed as

15 $${E}_{L}=\frac{{k}_{L}}{2}{\sum _{I=1}^{N}(\frac{{l}_{I}-{l}_{0}}{{l}_{0}})}^{2}.$$

The bending energy *E*_*B*_ is represented by the membrane curvature change between *θ*_*I*_ and ${\theta }_{I}^{0}$

16 $${E}_{B}=\frac{{k}_{B}}{2}\sum _{I=1}^{N}{\tan }^{2}(\frac{{\theta }_{I}-{\theta }_{I}^{0}}{2}).$$

The shape change is simulated through a penalty function. Γ_*s*_ is calculated by the relative area change with penalty function in order to ensure particle mass conservation. This function was defined as

17 $${{\rm{\Gamma }}}_{s}=\frac{{k}_{s}}{2}{(\frac{s-{s}_{e}}{{s}_{e}})}^{2}$$

where *s* is the particle area, *se* is reference value and *ks* is the areal elastic coefficient.

Liu *et al*.^51^ proposed the approach to model the inter-particles interaction energy *ϕ* as a Morse potential:

18 $$\varphi (r)={D}_{e}[{e}^{2\beta ({r}_{0}-r)}-2{e}^{\beta ({r}_{0}-r)}],$$

where *r* is the surface separation, *r*_0_ and *D*_*e*_ are, respectively, the zero force separation and surface energy, and *β* is a scaling factor controlling the interaction decay behavior. In present paper, this Morse potential was selected for its simplicity and realistic physical description of the particle-particle interaction and particle-wall interaction^33^. In our simulation, zero force distance is chosen as 1.0 lattice unit (0.5 μm) due to the An accurate representation of intercellular interaction force is not central to this paper, more detail of parameter determination is shown in Supplementary Information.

The motion of the particle was determined on the basis of the minimum energy principle.

19 $${f}_{i}=-\frac{{\rm{\partial }}(E+{{\rm{\Gamma }}}_{s}+\varphi )}{{\rm{\partial }}{r}_{i}}$$

where *f*_*i*_ is the membrane force, which can spread to the body force of the lattice.

### Benchmark

**Figure 9:**
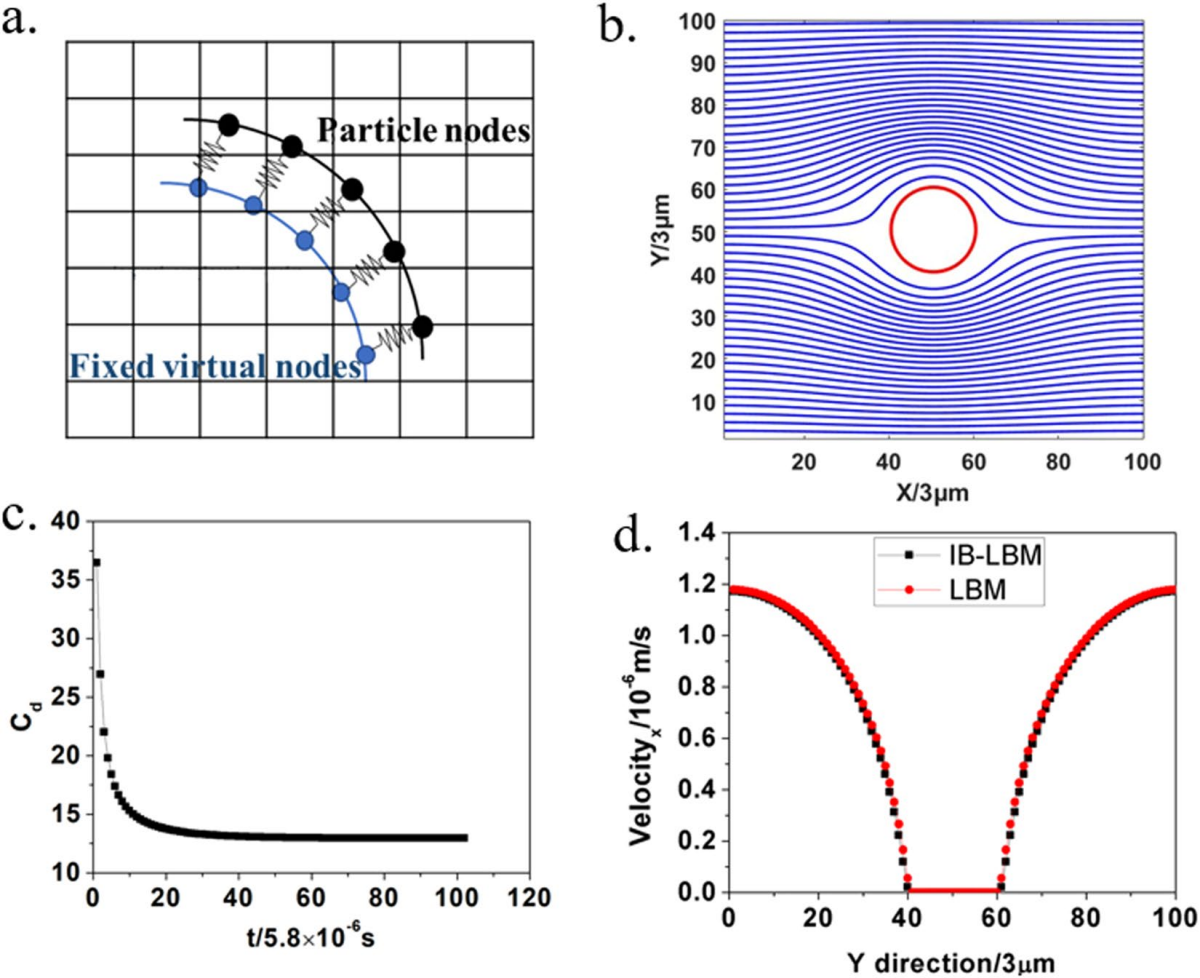
(**a**) The schematic of IB-LBM for flow passing over cylinder; (**b**) Flow field using IB-LBM; (**c**) The drag coefficient by IB-LBM simulation; (**d**) Vertical centerline velocity field distribution.

Referring to the work of Zhang *et al*.^33^, we have selected one typical benchmark case to verify the IB-LBM code of this paper for correct fluid-solid interaction capture. We model a circular ring by spring-network model in the fluid with a relatively large modulus. To mimic the solid objects in the flow field, each membrane node is linked to a fixed virtual node to hold the ring in original place and the fixed virtual nodes are not involved in the force distribution and position updating in IBM^33^, Fig. 9a. It is just like a hinge at both vertical ends of a cylinder to fix cylinder at the original place. According to Sangani and Acrivos^52^, the drag coefficient *C*_*D*_ of a square array of cylinders can be accurately approximated by

20 $${C}_{D}\equiv \frac{F}{\rho \nu U}=\frac{4\pi }{-\frac{1}{2}\,{\rm{l}}{\rm{n}}\,c-0.738+c-0.887{c}^{2}+2.038{c}^{3}+o({{\rm{c}}}^{4})}$$

For creeping flows and dilute arrays (c $\ll$ 1), here F is the drag force experienced by the cylinder and U is the mean flow velocity. Parameter c is the fractional area occupied by the cylinder in the domain

21 $$c=\frac{\pi {R}^{2}}{{L}^{2}}$$

where R is the cylinder radius and L is the domain size.

In our simulation, periodic boundary conditions are applied in both directions of the square domain to simulate flow passing over a square array of cylinders. A body force is 0.01 N/m^2^, applied to instead pressure boundary condition. We set R = 60 μm and L = 300 μm, kinematic viscosity = 1 × 10^−4^ m^2^/s and density of the fluid = 1 × 10^3^ kg/m^2^. The resulting drag force F = 8.81 × 10^−7^ N and mean flow velocity U = 6.81 × 10^−7^ m/s, the Reynolds number is R_e_ = 2UR/ν = 8.17 × 10^−7^, thus satisfying the creeping flow requirement. Figure 2b shows the flow field passing around the cylinder. The drag coefficient from IB-LBM simulation is ${C}_{D}^{IB-LBM}=F/\rho \nu U=12.944$, which is stationary quickly in Fig. 9c, while the analytical value of drag coefficient from Equation (20) was 12.286, the relative error is only 5.4%.

The velocity field of circular cylinder in the vertical centerline simulated by IB-LBM agrees well with LBM, as shown in Fig. 9d. The correlation of IB-LBM simulation with LBM simulation or theory analysis in velocity field and the drag force demonstrate the validation of the IB-LBM algorithm in this paper.

### AFM-based nanoindentation experiments

**Figure 10:**
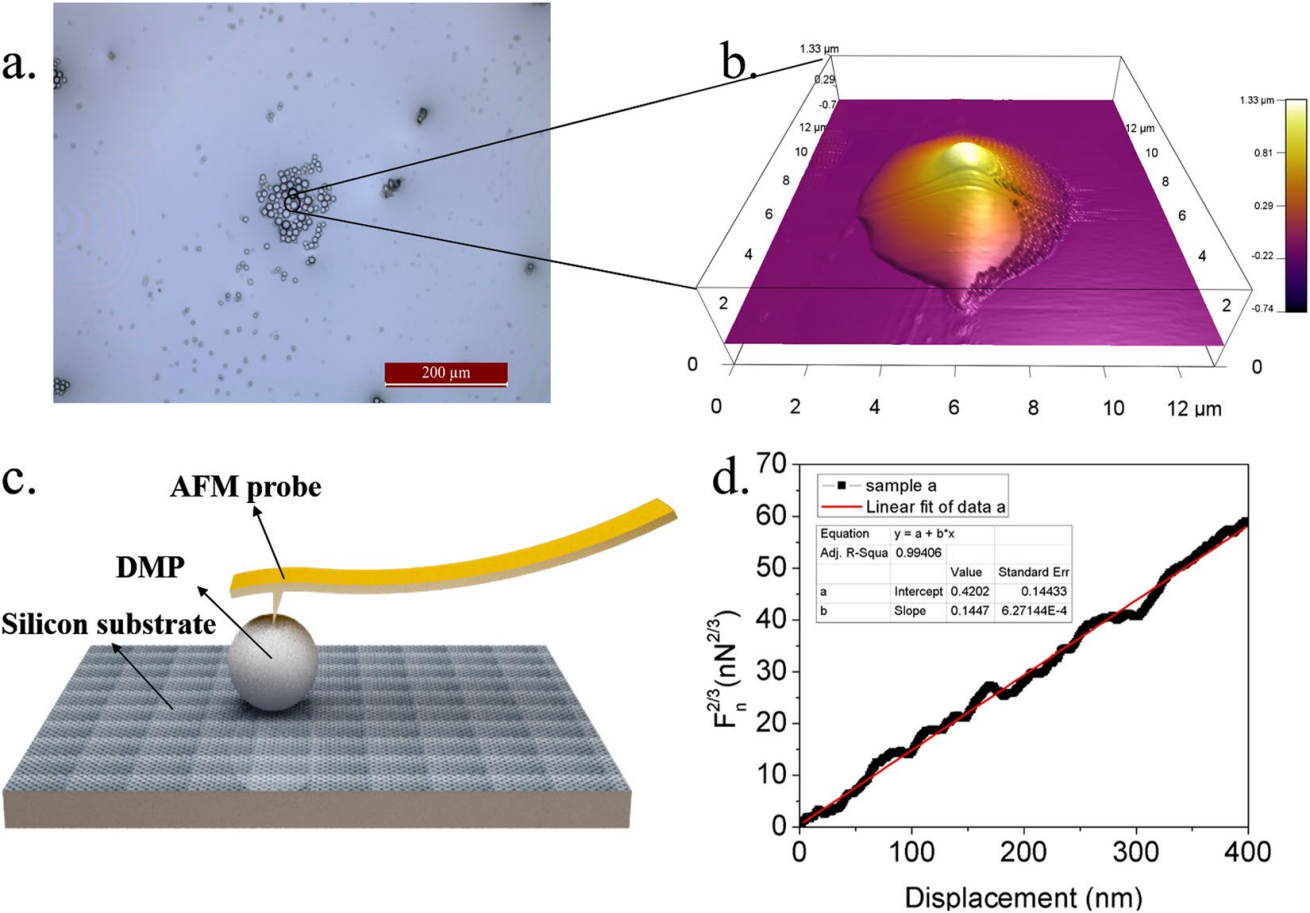
Experimental setup and microscale deformation measurement. (**a**) Microscopic image of DMP (SMG suspension) (**b**) 3D image of the particle. (**c**) A schematic diagram of the experimental setup. (**d**) The slope method of Hertz model fitting curve (one of the experiment data, others are shown in Supplementary Information).

The principle of the DMP elastic modulus measurement is to physically indent a particle with a probe and processing this force–indentation data using an appropriate model, such as Hertz model^53,54^. In this paper, size effect is not considered due to the complexity of the microsphere mechanical properties. The slope method of the Hertz model is selected for calculation of the elastic modulus of the DMP. DMP was prepared by coating on a silicon wafer, see Fig. 10a and elastic modulus was measured by indenting them with AFM tips. A spherical shape of DMP detected by AFM is shown in Fig. 10b, its diameter is about 1.3 μm and the particle surface has been covered by a layer of viscous fluid. The schematic diagram of the experimental setup was shown in Fig. 10c and the elastic modulus is calculated by the slope method of Hertz model, see Fig. 10d. The calculation method of the elastic modulus and the detail of the AFM-based nano-indentation experiments are shown in Supplementary Information.

### Determination of the spring-network model parameters

**Figure 11:**
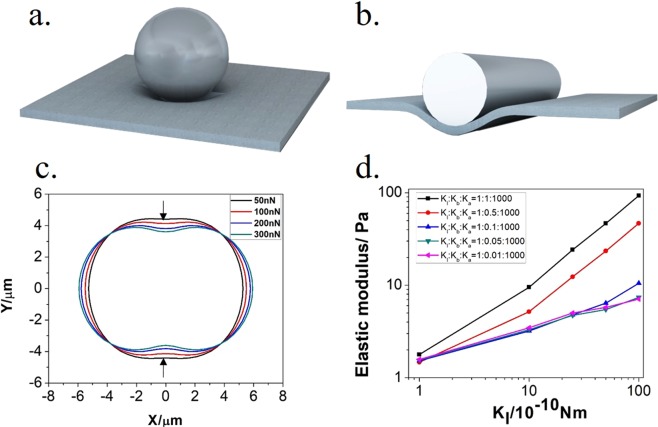
Nano-indentation simulation results. (**a**) A schematic diagram of particle deformation (3D) in nano-indentation experiment; (**b**) A schematic diagram of particle deformation (2D) in nano-indentation simulation; (**c**) 2D particle deformation in the nano-indentation simulation; (**d**) The relationship of elastic modulus and Kl & Kb.

AFM-based nanoindentation experiments give a guiding parameter of DMP’s elastic modulus, which is an important parameter in the simulation, see in Fig. 11a. The parameters of the spring network model can be determined by simulating the nano-indentation experiments (on the cylinder-cylinder or cylinder-substrate interface), shown in Fig. 11b. The force–indentation data can be processed by Hertz model. In order to get the proper particle elastic modulus, many nano-indentation simulations have been performed to determine the spring constants before each pore-scale simulation of DMP transport in channel, shown in Fig. 11c. The value of Kb and Ks follow the principle of K_l_:K_b_:K_s_ = 10:1:10000^50^, so we just adjust the value of K_l_ to the proper elastic modulus of the DMP model. The effect of K_b_ has been shown in Fig. 11d, K_l_ is more important than K_b_ in this case. Other parameters of the spring-network model and calculation method of elastic modulus are shown in Supplementary Information. When the value of K_l_ is 1.0 * 10^−9^ Nm, the elastic modulus is 3.20 MPa in the simulation, which is nearly consistent with the experiment value (3.22 MPa), here we don’t consider the elastic modulus of DMP size effect in this micro scale.

## Supplementary information

Supplementary Information to: Transport mechanism of deformable micro-gel particle through micropores with mechanical properties characterized by AFM
