# Supplementary material for: Transport mechanism of deformable micro-gel particle through micropores with mechanical properties characterized by AFM
Source: Sci Rep. 2019 Feb 5;9:1453. doi: 10.1038/s41598-018-37270-7 (PMC6363738; doi:10.1038/s41598-018-37270-7)
Supplement: Supplementary file 1 — Supplementary Information to: Transport mechanism of deformable micro-gel particle through micropores with mechanical properties characterized by AFM [file 41598_2018_37270_MOESM1_ESM.docx]

**Supplementary Information to:**

**Transport mechanism of deformable micro-gel particle through micropores with mechanical properties characterized by AFM**

Wenhai Lei^1^, Chiyu Xie^1,2^, Tianjiang Wu^3^, Xingcai Wu^4^ and Moran Wang1,[[1]](#footnote-1)†

^1^Department of Engineering Mechanics, Tsinghua University, Beijing 100084, China

^2^Department of Petroleum and Geosystems Engineering, University of Texas Austin, Austin 78705, USA

^3^Changqing Oilfield, PetroChina, Xi’an 710018, Shaanxi, China

^4^Research Institute of Petroleum Exploration & Development of PetroChina, Beijing 100083, China

**Supplementary Note 1. Experimental details**

SMG is one of the typical DMP suspensions and has been widely tested in gel treatments in oilfields^1^. The DMP surface was first scanned to identify the shape profile and then indented to measure the deformation response. Spherical indenters are used to reduce the potential for penetration and rupture. In this paper, diamond-like-carbon (DLC) coated silicon AFM tips DCP10 (TipNano, Estonia) were assembled. Hertz-based equations were developed specifically for solid-to-solid contact of elastic, isotropic materials where the contact region was negligible compared to the bodies themselves.

| $f_{sphere}=\left(\frac{4}{3}\frac{E}{1-\nu^{2}}\sqrt{R}\right)\delta^{\frac{3}{2}}$, | (A1) |
| --- | --- |
| $\left(f_{sphere}\right)^{\frac{2}{3}}=slope\times\delta$, | (A2) |
| $slop=\left(\frac{4}{3}\frac{E}{1-\nu^{2}}\sqrt{R}\right)^{\frac{2}{3}}$, | (A3) |
| $E=\frac{3}{4}\frac{\left(1-\nu^{2}\right)}{\sqrt{R}}slop^{\frac{2}{3}}$, | (A4) |

where f_sphere_ is the pressure exerted by the probe on the particles, E is the elastic modulus, ν is the Poisson's ratio, due to the complexity of the soft matter, the Poisson's ratio is regarded as 0.5^2,3^ in this paper, R is the probe radius, the typical curvature radius of the tip is about 100 nm as provided by the manufacturer, δ is the depth of the indentation. By linear fitting in Eq.(A3), the slope of the $\delta\text{\textasciitilde}\left(f_{sphere}\right)^{\frac{2}{3}}$curve can derive the elastic modulus by Eq.(A3-A4). $\delta\text{\textasciitilde}\left(f_{sphere}\right)^{\frac{2}{3}}$ curves are shown in fig. A1.


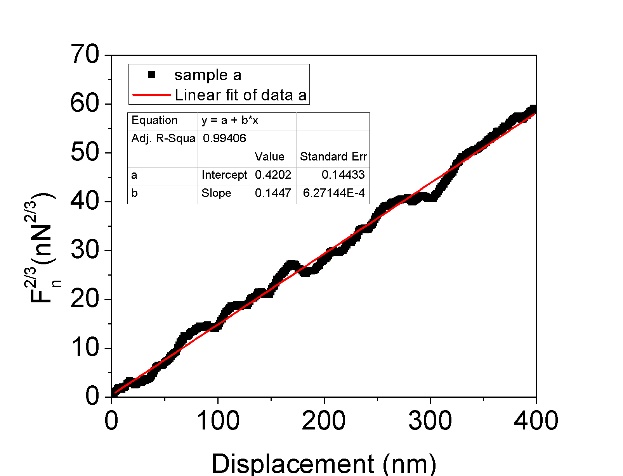

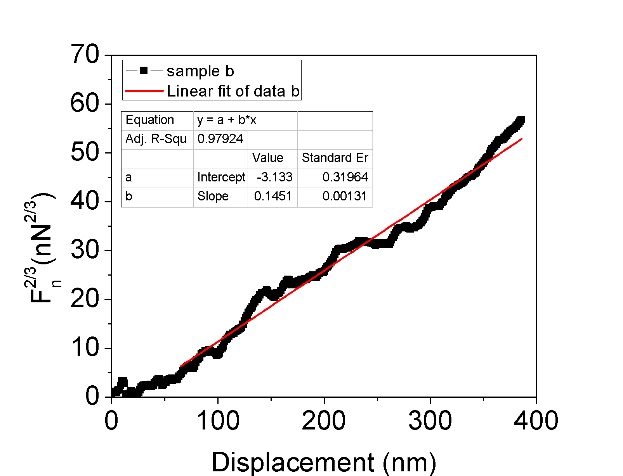

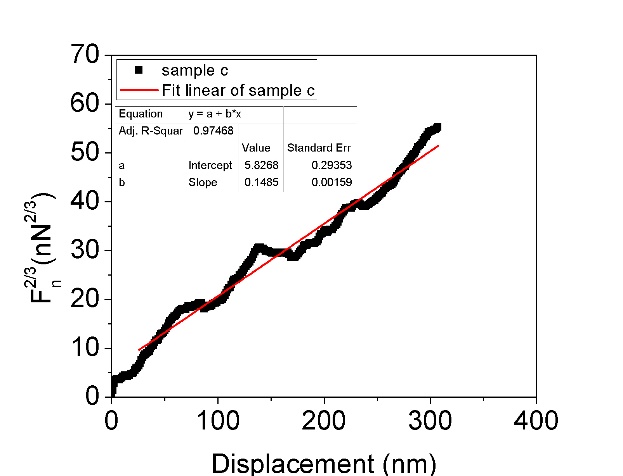

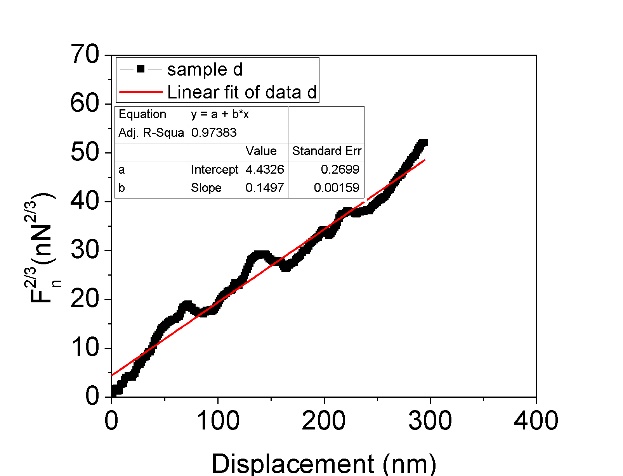

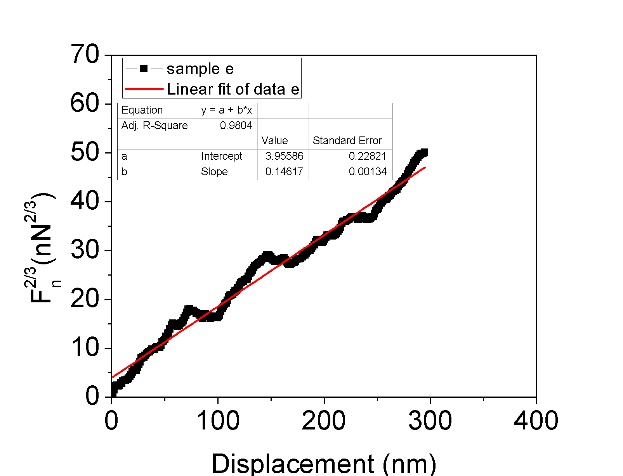

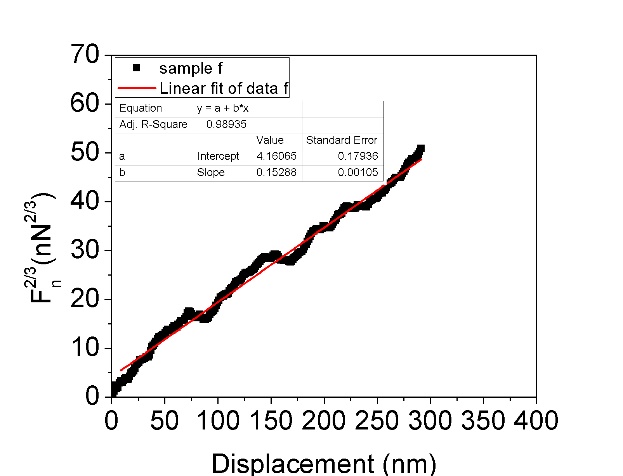


b.

a.

l.

k.

h.

g.

f.

e.

d.

c.

b.

a.


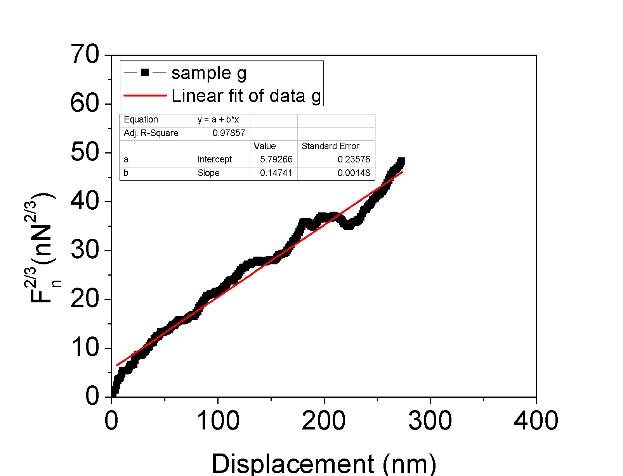

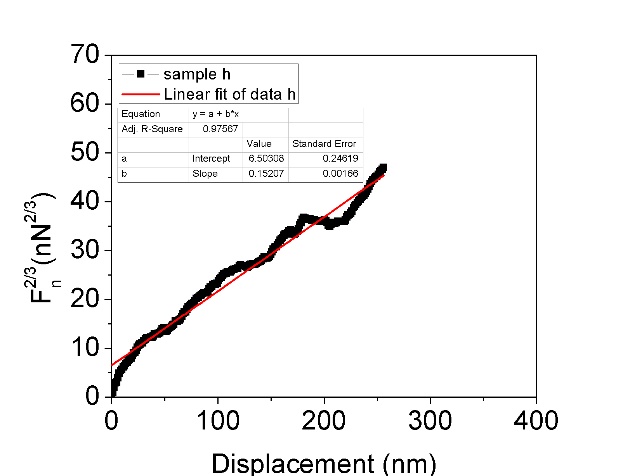
 .
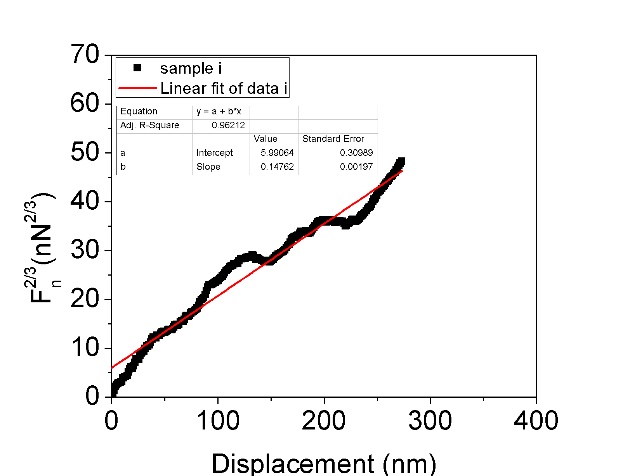

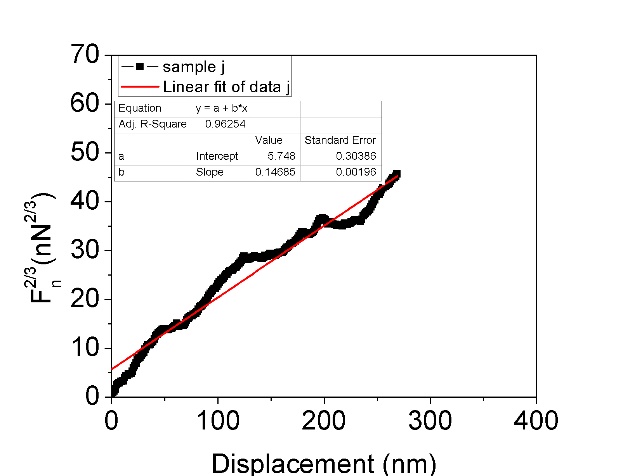


j.

i.


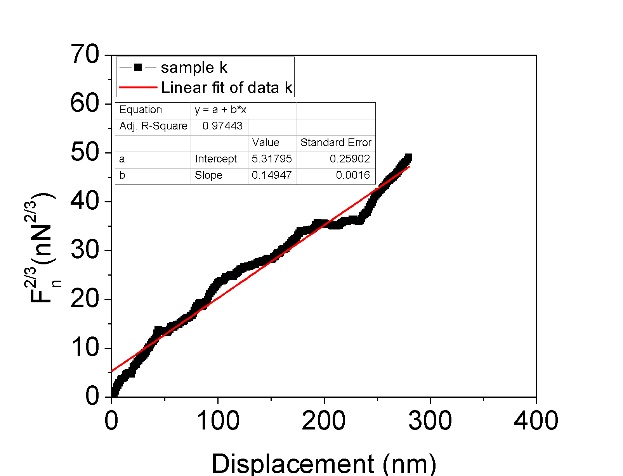

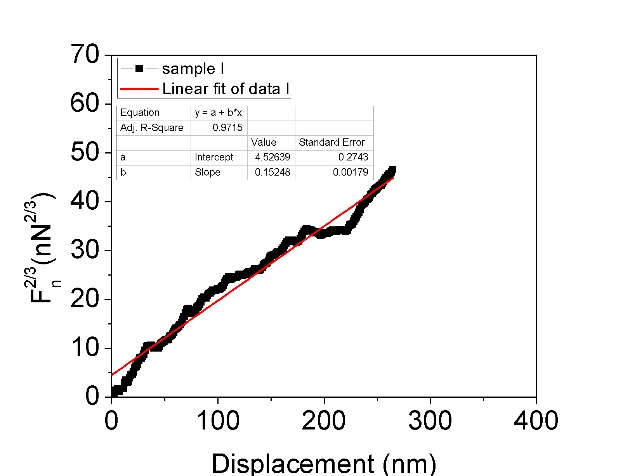


k.

l.

**Figure A1. Experiments results of** $\delta\text{\textasciitilde}\left(f_{sphere}\right)^{\frac{2}{3}}$**curve.** Experiments (a-l) measure the same particle.

The experiments results are shown in Table A1. The standard deviation of this experiments has two parts, standard deviation of each nanoindentation experiment fitting to get elastic modulus $\sigma_{B}$ and standard deviation of all elastic modulus to get final elastic modulus$\sigma_{A}$. The final standard deviation can be expressed as

| $\begin{matrix} \sigma=\sqrt{\sigma_{A}^{2}+\sigma_{B}^{2}} \\ \sigma_{A}=\sqrt{\frac{\left(x_{i}-\frac{\sum x_{i}}{n}\right)^{2}}{n}}; \sigma_{B}=\max\sigma_{i} \end{matrix}$. | (A5) |
| --- | --- |

Consequently, the elastic modulus of the SMG particle is 3.22±0.095 MPa, shown in Table A2. The standard deviation $\sigma_{B}$ can be ignored in the experiment, compared with the standard deviation$\sigma_{A}$.

Table A1. Elastic modulus of the SMG particle calculated by Hz model

| Sample | E(MPa) | $\sigma_{i}$ | R-square |
| --- | --- | --- | --- |
| a | 3.10 | 8.83×10^-4^ | 0.994 |
| b | 3.11 | 2.67×10^-3^ | 0.979 |
| c | 3.22 | 3.57×10^-3^ | 0.975 |
| d | 3.26 | 3.57×10^-3^ | 0.974 |
| e | 3.14 | 2.76×10^-3^ | 0.980 |
| f | 3.36 | 1.94×10^-3^ | 0.989 |
| g | 3.18 | 3.20×10^-3^ | 0.979 |
| h | 3.34 | 3.80×10^-3^ | 0.976 |
| i | 3.19 | 4.92×10^-3^ | 0.962 |
| j | 3.17 | 4.88×10^-3^ | 0.963 |
| k | 3.25 | 3.60×10^-3^ | 0.974 |
| l | 3.35 | 4.66×10^-3^ | 0.972 |

Table A2. Elastic modulus of the SMG

| $\overline{E}$(MPa) | $\sigma_{A}$ | $\sigma_{B}$ | $\sigma=\sqrt{\sigma_{A}^{2}+\sigma_{B}^{2}}$ |
| --- | --- | --- | --- |
| 3.22 | 9.01×10^-2^ | 4.92×10^-3^ | 9.50×10^-2^ |

**Supplementary Note 2. Simulation details**

The parameters of DMP model are selected as table 1, the node number n is fixed as 60, and the particle diameter is 10μm. Intercellular interaction strength D_e_, scaling factor β and zero force distance r_0_ are acquired from reference^4^. In our simulation, zero force distance is chosen as 1.0 lattice unit (0.5μm) due to the limitation of the grid size and the immersed boundary treatment employed in the computational scheme, which is larger than zero force distance (~14nm) according to Neu and Meiselman (2002)^5^. This distance is relatively large compared to experimental data and the scheme could be improved by adopting a smaller lattice grid and/or the adaptive mesh refinement technology^4^. A cut-off distance is adopted to improve the computational efficiency as is typical in molecular dynamics simulation. The scaling factor β is adjusted so that the attractive force beyond the cut-off distance r_c_ =5 lattice units (2.5μm) is neglected (β= 3.84μm^-1^). The value of K_b_ ^1^and K_s_ follow the principle of K_l_: K_b_: K_s_ = 10:1:10000^6^. We just adjust the value of K_l_ to the proper elastic modulus of the DMP model.

In indentation simulation, the force is applied on the three nodes of the particle two sides and the range is about 0-300nN considering the experiments, so the probe radius is about 0.75μm, which is much less than the particle diameter and treating the indentation simulation as a “quasi-static” problem by each step of the system to be stationary. Indentation is simulated at a series of forces to get the correlation depths. The elastic modulus is calculated by Hertz model of cylinder indentation (on the cylinder-cylinder or cylinder-substrate interface) in Eq.(B1), we get the different elastic modulus of different K_l_ values in table B2. $\sigma_{i}$ is the standard deviation of the elastic modulus calculated by the slope method by Hertz model in the indentation simulation.

| $f_{cylinder}=E\frac{\pi\delta}{4\left(1-\nu^{2}\right)}$ | (B1) |
| --- | --- |

Table B1. The parameters of the DMP model

| Parameter | Value | Parameter | Value |
| --- | --- | --- | --- |
| n | 60 |  | 10μm |
| l_0_ | 0.52μm | dx | 0.5μm |
| dt | 8.3*10^-9^s | β | 3.84μm^-1^ |
| K_b_ | 0.1*K_l_ | S_0_ | 0.79μm^2^ |
| K_s_ | 1000*K_l_ | D_e_ | 5.2*10^-8^μJ/μm^2^ |
| r_0_ | 0.5μm | r_c_ | 2.5μm |

Table B2 .Different elastic modulus of different K_l_ values

| No. | Value | Elastic modulus | $\sigma_{i}$ | R-square |
| --- | --- | --- | --- | --- |
| 1 | 1.0*10^-10^Nm | 1.53MPa | 0.153MPa | 0.952 |
| 2 | 1.0*10^-9^Nm | 3.20MPa | 0.285MPa | 0.962 |
| 3 | 2.5*10^-9^Nm | 4.77MPa | 0.234MPa | 0.988 |
| 4 | 5.0*10^-9^Nm | 6.39MPa | 0.287MPa | 0.990 |
| 5 | 1.0*10^-8^Nm | 10.5MPa | 0.206MPa | 0.998 |

## References

1 Wu, X. *et al.* in *SPE Latin America and Caribbean Petroleum Engineering Conference* (Society of Petroleum Engineers, Buenos Aires, Argentina, 2017).

2 Radmacher, M., Fritz, M., Kacher, C. M., Cleveland, J. P. & Hansma, P. K. (Elsevier, 1996).

3 Costa, K. D. Single-cell elastography: probing for disease with the atomic force microscope. *Disease markers* **19**, 139-154 (2004).

4 Zhang, J., Johnson, P. C. & Popel, A. S. Red blood cell aggregation and dissociation in shear flows simulated by lattice Boltzmann method. *Journal of biomechanics* **41**, 47-55 (2008).

5 Neu, B. & Meiselman, H. J. Depletion-mediated red blood cell aggregation in polymer solutions. *Biophysical journal* **83**, 2482-2490 (2002).

6 Tsubota, K.-i. & Wada, S. Effect of the natural state of an elastic cellular membrane on tank-treading and tumbling motions of a single red blood cell. *Physical Review E* **81**, 011910 (2010).

1. ^†^ Corresponding author; Email: mrwang@tsinghua.edu.cn [↑](#footnote-ref-1)
